# Buying best value health care: Evolution of purchasing among Australian private health insurers

**DOI:** 10.1186/1743-8462-2-6

**Published:** 2005-03-31

**Authors:** Sharon Willcox

**Affiliations:** 1 Victorian Department of Human Services, 555 Collins Street, Melbourne, 3000 Australia; and La Trobe University, Bundoora, 3086, Australia

## Abstract

Since 1995 Australian health insurers have been able to purchase health services pro-actively through negotiating contracts with hospitals, but little is known about their experience of purchasing. This paper examines the current status of purchasing through interviews with senior managers representing all Australian private health insurers. Many of the traditional tools used to generate competition and enhance efficiency (such as selective contracting and co-payments) have had limited use due to public and political opposition. Adoption of bundled case payment models using diagnosis related groups (DRGs) has been slow. Insurers cite multiple reasons including poor understanding of private hospital costs, unfamiliarity with DRGs, resistance from the medical profession and concerns about premature discharge. Innovation in payment models has been limited, although some insurers are considering introduction of volume-outcome purchasing and pay for performance incentives. Private health insurers also face a complex web of regulation, some of which appears to impede moves towards more efficient purchasing.

## Background

The last decade has witnessed a revolution in payment arrangements for public hospitals in Australia. Victoria was the first state to introduce casemix using diagnosis related groups (DRGs) as the basis of paying for inpatient care in public hospitals in 1993. Within 3 years, all other states except New South Wales had followed suit [1]. The widespread adoption of DRGs to replace historical budgets resulted in measurable gains in productivity as public hospitals became accountable for delivery of specified outputs [2]. DRGs group together patients with similar expected resource utilization, shifting the incentive from purchasers to health providers to manage the components of care more efficiently.

Contemporaneously, the Commonwealth Government legislated in 1995 to enable contracting between insurers, hospitals and doctors, arising from concerns about declining private health insurance membership fuelled by consumer dissatisfaction at insurance co-payments [3,4]. Commonwealth regulation of contracting arrangements under the *National Health Act 1953 *requires that contracts between hospitals and insurers be described on the basis of casemix episodic payments using Australian National DRGs (AN-DRGs). However insurers and hospitals have flexibility as to the actual structure of the payment mechanism used in such contracts.

In introducing the 1995 contracting reforms, the Commonwealth Minister proposed that one of the central objectives was to transform private health insurers from "passive bill payers" into active purchasers of services for their members [5]. Health purchasing has been defined as "buying the best value for money services to achieve the maximum health gain for those most in need" [6]. However this definition is from a study of the experience of the British National Health Service and the reference to people 'most in need' may be less relevant to the role of private sector purchasers. In a review of major health service purchasers in the United States, 'value purchasing' was defined as "an organized attempt by a private- or public-sector purchaser to ensure quality and to improve health outcomes, as well as negotiating prices, as an explicit part of its health care buying strategy" [7]. The simpler version of this definition was that value purchasing involved "getting the best care for the best price".

Australian private health insurance provides access to doctor of choice and private hospitals, together with covering ancillary services such as physiotherapy and dental care. In turn, the public Medicare program provides free access to public hospitals and subsidized access to medical services and pharmaceuticals. The issue of whether private health insurance duplicates or supplements Medicare has been considered in several major reviews, including by the Industry Commission and the Organization for Economic Co-operation and Development [8,9]. The lack of consensus on the desired role of private health insurance in the context of a universal public insurance program complicates the design of the appropriate regulatory framework for private health insurance.

Private health insurance is tightly regulated by the federal government under the *National Health Act 1973*, although the legislation itself lacks a set of clear policy objectives to provide a coherent rationale for regulation [9]. The purchasing role of health insurers is significantly shaped and potentially constrained by regulatory requirements including:

• Clinical autonomy – health insurance contracts are required to maintain the medical practitioner's professional freedom within the scope of accepted clinical practice;

• Contracting flexibility – health insurers are prohibited from refusing to contract with hospitals on the basis of the number of hospital beds, the range of hospital treatments or the hospital ownership arrangements;

• Benefit mandates – all health insurance products must provide coverage for palliative care, psychiatric services and rehabilitation;

• Second tier default benefits – health insurers are required to pay benefits equal to 85% of average contracted benefits to private hospitals with which they do not contract; and

• Reinsurance – the costs of high service users and the elderly are redistributed and equalized across all health insurers.

While the first three of these requirements directly limit the scope of acceptable contracting by health insurers, the default benefits and reinsurance arrangements are generally perceived to be equally, if not more, significant in influencing purchasing behavior. Interestingly, the legislation is silent on 'positive' criteria for purchasing and does not stipulate use of methods such as cost-effectiveness, or require insurers to consider factors such as quality, clinical outcomes, value-for-money or efficiency, in entering contracts with hospitals and medical professionals.

At the macro-regulation level, two other factors influence the purchasing environment of private health insurers. The 1999 introduction of a 30% tax rebate for private health insurance creates a clear interest by the Commonwealth Government in whether health insurers are purchasing services efficiently. However, recent boosts in membership following the introduction of lifetime community rating create a countervailing pressure to maintain the value proposition of private health insurance through unrestricted choice and access to private services.

The evolution of purchasing among Australian health insurers subsequent to the 1995 legislation has received no direct consideration in the academic literature. Since 1999 the Australian Competition and Consumer Commission (ACCC) has produced regular reports that examine contracting by health insurers to assess the extent of any anti-competitive practices by either health insurers or health providers which may have a detrimental effect on consumers [10]. While these reports constitute a rich resource of the views of stakeholders on contracting, they do not directly examine how health insurers are attempting to meet the purchasing challenge of 'getting the best care for the best price'. Industry conferences, together with trade publications, provide some partial and tantalising glimpses of the evolving purchasing behavior of health insurers, but again do not provide a comprehensive picture.

Accordingly, a study was designed to address this gap in our knowledge of whether Australian health insurers are buying best value health care for their members. Semi-structured interviews were conducted with key decision-makers across all Australian private health insurers to elicit their views on the evolution of purchasing across the dimensions of quality and coverage of new technology ('best care') and payment models ('best price'). This paper reports the findings relating to payment models. A future paper will provide full details of the study methodology, together with reporting the 'best care' findings on quality and coverage.

## Results

Health insurers were initially asked to describe their current payment models including the perceived benefits and risks of different models, and how their approach to payment policy had changed over time. This led to more expanded discussions on the challenges associated with purchasing private health services including the regulatory framework for health insurance, relationships with private hospitals and issues with contracting. The findings from these interviews are presented under three main themes.

First, the purchasing environment is examined from the perspective of insurers, including their views on options for contracting, supplier-induced demand, gap payments, the impact of regulation on contract negotiations, and their relationships with private hospitals. The second section describes the current status of payment models across the industry including issues and barriers. Finally, the third section identifies examples of innovation in purchasing by private health insurers with a view to highlighting potential future developments in payment models.

### Purchasing environment

#### Selective contracting

Health insurers were acutely conscious that government policy changes to encourage higher levels of health insurance membership had a direct influence on public perceptions about the value of insurance, and hence impacted on their ability to exercise greater purchasing discipline. A critical dimension of pro-active purchasing of health services involves the ability to select providers on the basis of criteria such as quality and efficiency in service delivery. However, this implies some restriction in choice of service providers for members, a difficult message as noted by one insurer:

"Funds tend to have a philosophy of wanting to offer a wide a choice as possible, and at this point in time, after the initiatives of lifetime health cover and all that entails, and the 30% rebate, this is not a good time to be selectively contracting, because it means that you may restrict access."

In addition to anticipated consumer resistance, some insurers believed that previous selective tendering processes by other insurers had generated "a lot of angst" and "mistrust" among private hospitals. One insurer noted that the concept of selective contracting was almost a "taboo" topic. Moreover, insurers argued that government regulation, requiring insurers to pay a second-tier default benefit to hospitals that did not win contracts (equal to 85% of contracted benefit payment), undermined their negotiation ability and resulted in "propping up" private hospitals.

"So second tier and portability are going to make the competitive tension very difficult in the industry, very difficult....It needs some sort of competitive tension to, if you wish to direct your business or have some sort of preferential arrangements."

Insurers also noted that the potential for some "iconic hospitals" to not gain contracts had major public and political risks as these disenfranchised hospitals used the media to argue against the merits of contracting decisions by insurers. Despite general reservations about the difficulty in refusing to contract with certain hospitals, two insurers gave examples of situations where they had confronted proprietors planning to open new facilities to inform them that they would not receive insurance benefits. One case was based on the insurer's view that there was an adequate supply of psychiatric services in a geographic region, while the other case related to concerns about establishing a cardiac catheterisation laboratory in a small hospital with no immediate access to supporting intensive care or coronary care units. It appeared in both cases that this pre-emptive positioning by insurers had halted further development, although private hospitals could have simply ignored this threat, established the services and proceeded to claim second tier default benefits.

#### Supplier-induced demand

One of the reasons why insurers expressed concern about not being able to exercise greater purchasing power through selective contracting was because they believed that providers could often generate demand for unnecessary health care services. This view was expressed most succinctly by one insurer:

"Access equals demand. You open it and it'll be full and it'll be new demand."

However another insurer challenged this view, citing the spare capacity in many private hospitals and examples of private hospitals that had experienced financial difficulty due to low occupancy. Most insurers expressed reservations about the lack of government planning or quality controls for new hospitals, singling out the growth in day procedure facilities that they suggested were "jumping up everywhere".

The lack of success by insurers in having medical services included in their contracts with private hospitals (the 'practitioner agreements' provisions in the 1995 legislative amendments) has also stymied their ability to influence service provision levels. Insurers noted the potential for over-servicing, with one respondent arguing that loose arrangements between private hospitals and doctors, together with the lack of a "strong teaching environment" created the preconditions in which inappropriate or unnecessary care could be provided. Insurers noted that entrepreneurial claiming behavior by some health providers required constant vigilance in benefits management.

"The incentives that exist to generate income are quite strong and as a result, you get some pretty poor behavior, sometimes fraudulent, but certainly creative as to how you would generate income as an individual... So we have a whole industry now of what we call benefits limitation, benefits management."

#### Consumer co-payments

Since 2000, government regulation has required that private health insurance for medical services related to a hospital admission be provided on either a 'no gap' or 'known gap' basis [11]. While there is no similar legislative requirement on hospital costs, interviews confirmed that the widespread industry practice was that hospital contracts were negotiated to minimize consumer co-payments. As with selective contracting, this was justified on the basis that co-payments would "water down the value proposition" of private health insurance. Only one major insurer had a policy of explicitly using hospital co-payments to "promote competition" and to "send a message to consumers" to reassess the medical necessity of their use of hospital services.

Generally, insurers noted that even members who had chosen to purchase cheaper insurance products, with front-end deductibles or specified hospital co-payments, often became upset when faced with having to meet these costs themselves on admission to hospital.

Perceptions of public aversion to insurance co-payments meant that most insurers were reluctant to establish a system of tiered hospitals, that might encourage members to use preferred hospitals with zero co-payments compared to other hospitals with higher co-payments. Insurers argued that their members would view this as discriminatory and that the complexity of such a payment system would make health insurance more confusing. While some insurers distinguished between 'participating' and 'non-participating' hospitals, this tended to be related to insurers largely contracting with hospitals in the service area of their members.

#### Impact of regulation on purchasing ability

Insurers often referred to the complex web of Commonwealth Government regulation of their industry and its impact on their purchasing behavior.

"Purchasing is certainly not easy with any sector. It's never quite black and white; there's a whole range of other issues than price. So it's not like you're going out and buying a simple can of baked beans. But it's certainly made a whole lot more difficult when you've got legislation that actually prevents you from doing a good job of purchasing."

The most frequently cited examples of problems with regulation related to second tier default benefits, reinsurance, outreach services and prostheses. Of these, the regulation of outreach services provides a useful illustration of the difficulty in reconciling conflicting policy objectives. Government regulation has established a reinsurance pool that redistributes the costs of particular groups across all insurers, in order to support the principle of community rating and ensure that insurers with higher claiming members do not face a spiralling cycle of adverse selection. However the reinsurance arrangements only apply to services provided under hospital insurance products, not ancillary products, meaning that services not directly associated with a hospital admission are not eligible for inclusion in the reinsurance pool. To overcome the consequential disincentive to provide effective substitutes to hospital-based care, the Commonwealth Government enacted further regulation establishing a tightly prescribed framework under which outreach services could be provided.

However insurers were critical of the resulting approval process needed for outreach services, noting:

"And it just seemed to be completely an aberration of going back to, God knows how many decades ago, where getting out of the hospital walls, you've got to have this huge bureaucratic process just to get a home-based care program, which has been running maybe in the public system for years."

Another problem raised by insurers was that the outreach legislation only recognizes outreach programs that are provided by hospitals, whereas insurers may want to purchase these services directly from community-based providers.

#### Relationships with the private hospital sector

Insurers often commented on the mutually dependent relationship they had with private hospitals, arguing that they were "working for the common cause". One insurer linked the financial viability of private hospitals and health insurers as follows:

"Value for money in a health fund product is important, so if you have an argument with a hospital that says: you get more business if we have more customers, if you charge us too much more, then we can't keep our customers. So we can actually improve your profitability and keep you full, but only if we don't get gouged. So make profit off volume, by all means, but don't make profit off individual patients, because the number of patients off who you'll be able to make a profit will diminish rapidly over a short period of time. So we have to live symbiotically here."

The importance of building mature relationships with private hospitals was also raised. It was argued that this required insurers to have "credibility" and be viewed by private hospitals as "member and patient focussed, not just money focussed". Credibility included having the strength of being principled, that is, "we actually do as we say we'll do", but also being open to discussion with private hospitals. In turn, insurers noted that they were more prepared to consider funding new service models if they had "faith" in particular private hospitals and there was "goodwill on both sides".

One insurer stressed that contracting was only part of their relationship with private hospitals, which needed to be maintained over time irrespective of whether they were in or out of contract with individual hospitals. Another insurer lamented the focus on "perpetual contract negotiation" as detrimental to the ability to build strong relationships, arguing that it would be preferable to move to long term contracts. Most insurers spoke of annual contract negotiations with providers, linked to the inability to accurately estimate future cost growth.

### Current status of payment models

**Figure 1:**
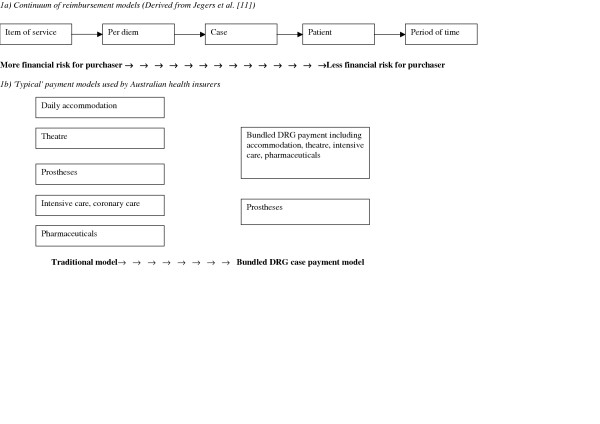
Payment models for hospital services.

Payment models can be classified along a continuum according to the unit of reimbursement [12]. Figure 1 illustrates that purchasers face greater financial risk with relatively unbundled payment models, such as fee-for-service or daily payments as providers have an incentive to increase the level of outputs. In contrast, financial risk is transferred away from purchasers to providers through payment models such as capitation models (where the unit of reimbursement is the patient) and case payments or episodic models. (see figure 1)

Interviews revealed that there is currently considerable variation across private health insurers as to the payment models used as the basis of hospital contracts. Figure 1 also illustrates the two 'typical' payment models used by health insurers for most hospital services.

Most insurers still rely strongly on a 'traditional' payment model that involves separate payments for each of the components of hospital care including accommodation, theatre, prostheses, pharmaceuticals and intensive care unit (ICU) costs. Insurers usually refer to this as a per diem payment model. It is based on classifying patients according to the type of medical service provided using Medicare Benefit Schedule (MBS) items, with payment step-downs to encourage shorter lengths of stay. When viewed against the payment model continuum, it can be seen that this traditional model is actually more similar to an item of service payment model than a true per diem model.

The second typical payment model is the 'bundled DRG case' payment model where most hospital admission costs are bundled into a single payment. Currently only two major insurers (representing about one-quarter of the insurance market) are using an almost fully bundled DRG model (with bundling of theatre but separate payments for prostheses) for the majority of their acute episodes. Two other major insurers (representing about one-half of the market) had undertaken substantial developmental work and were planning significant expansion in their use of DRGs. However, one crucial difference in the use of DRGs between the private and public sectors is that medical costs are specifically excluded in the private sector, with DRGs only applying to non-medical services.

Within these two 'typical' payment models, there is scope for numerous permutations. For example, some insurers relying largely on per diem payments still operate a more bundled case payment model for a limited number of conditions with relatively predictable costs, such as obstetrics, dialysis and endoscopy. One insurer referred to bundling in the cost of low cost prostheses in a predominantly per diem payment model. Of the two insurers using a bundled DRG case payment model, one bundled in ICU costs.

#### Attitudes and barriers to adoption of DRGs

Insurers, irrespective of their payment model, commented on some of the advantages of using DRGs relative to the predominant per diem model including:

• "DRGs are the only thing that is statistically coherent and recognizes all elements of care and you really can't say 'we're different'."

• "And there's considerable scope, we believe, something like a 30% scope for reduction of length of stay in the private sector."

• "The hospital has an opportunity to do more, to innovate and to make things more cost-effective, in the way they can and they gain by that. And we gain by a greater predictability."

• "I think it's crazy when you benchmark by DRG and then pay by per diem. I think there has to be consistency between your benchmarking and your payment model."

• "So our aim was to try and get the hospitals onboard, to try and share the risk, that's our big basis behind our case payment, share the risk."

The relatively slow progress in uptake of DRGs relative to the public sector was attributed to a wide range of factors including limited data and poor understanding of the cost structures of private hospitals, the lack of familiarity and understanding of DRG based payment models, resistance from the medical profession, and concerns about potentially premature discharge.

Of these, the lack of good costing systems was most frequently mentioned, with insurers noting that "the industry is very much historically based". Accordingly, some insurers referred to using "price weights" based on historical prices, rather than cost weights. Most insurers were keen to get greater access to private hospital cost data. However a dissenting view was offered by one insurer who cautioned that "the more you get involved in their cost structure, the more you become a funder". This insurer was hesitant about moving from a purchasing role to a situation where insurers might be regarded as being responsible for the financial sustainability of contracted private hospitals. Insurers also suggested that some private hospitals did not have the capacity to assess properly how efficiency gains flowing from the use of DRGs could positively impact on their operating margins.

"And what we've been trying to do is get the facilities to look at their margins, rather than their revenue. Their pure revenue increases. And not many of them are at that level of sophistication. Because we believe and can show some of them that if they do things differently, they can actually get a greater margin, rather than just increases in pure revenue. But they don't think like that, many of them."

Insurers reported that providers sometimes equated the use of DRGs with insurers taking on an undesirable 'managed care' role.

"So even to bring in a casemix funding system which is already out there, and has already been in the public system for years, we got pushback from the AMA saying: 'that's managed care', because it was imposing a length of stay through the inlier type separation. And those sorts of barriers are just so alive and well because of the nature of the power that the doctor has in the private sector."

The medical profession has long resisted entering contracts with health insurers [11] and this insurer's comments highlight the power of doctors to influence other payment arrangements between insurers and hospitals. From the perspective of insurers, the exclusion of medical costs from DRGs in the private sector is likely to reduce the potential efficiency gains and may partially explain the slower adoption of DRGs in the private sector.

Insurers who had introduced DRGs considered that they had done so in a cost neutral manner, without clawing back the ensuing efficiency savings made by private hospitals. This was motivated partially by concerns about avoiding the 'quicker and sicker' phenomenon, often associated with the use of DRGs.

Several insurers noted that there was now considerable interest, and indeed pressure, from many private hospitals for the more uniform adoption of DRGs across private health insurers.

"I think they want homogeneity. If you look from a provider's perspective.... the thing that you hate the most is that each fund has a different methodology about how you submit your claims and how you get paid... So their perspective is, if it standardizes along the lines of a casemix type structure, I think their view, and the Australian Private Hospitals' Association view, is that that would be a good thing. If they can achieve some back office efficiencies, then that would help them in the management of their business and it would also assist them into getting into e-claiming. That's where they want to get into. It has huge efficiencies for them, and for us, but that needs, largely, a similar contracting, purchasing base."

### Innovation in purchasing by health insurers

Bundled DRG case payments are one mechanism by which purchasers can encourage providers to seek new approaches to delivering care more effectively, through transferring risk to providers. However, Figure 1 shows that there is potential beyond case payments to encourage greater allocative efficiency through paying providers on the basis of capitation or episodic payments.

Insurers who have experience of using DRGs are more likely to become aware of some of the limitations of DRG case payments. These include ensuring that providers take responsibility for pre-admission and post-discharge services, or the fact that DRG case payments still contain inherent incentives for the production of unnecessary hospital admissions. In response, these insurers might move towards greater innovation in purchasing by trialling payment models that require providers to assume financial responsibility for an expanded scope of care.

In fact, interviews for this study found that most innovation in purchasing was concentrated in one of the two major insurers that had already implemented a bundled DRG case payment model. Table 1 provides an outline of four examples of innovative purchasing developed by this insurer. Two of these examples, episodic management units and the members extended care arrangements, involve the provider assuming greater responsibility for services over an extended period of time relative to the narrow DRG case definition. The capitation model involves the psychiatric services provider receiving a fixed payment for each insured member, irrespective of the actual services provided. (see Additional file 1)

While all these innovative purchasing models originated with one insurer, several other insurers had followed its lead in adopting the same capitation model for psychiatric services in one state. At interview, the second major insurer using a bundled DRG case payment model did not outline similar payment models focussed on transferring risk, but instead emphasized the importance of clear specification of required quality parameters in hospital contracts.

Insurers were also asked about their views on innovative payment models that explicitly link purchasing to quality. This might include pay for performance frameworks, or only purchasing services if hospitals undertook at least the minimum volume recommended to enhance safe patient outcomes.

#### Volume-outcome purchasing

Several insurers had undertaken exploratory work on the potential introduction of 'volume-outcome' purchasing. In particular, one insurer was cautiously beginning to apply this framework in purchasing cardiac, neurosurgery and obstetric services. As one insurer noted:

"If you've got good volume, you don't necessarily have good quality, but if you've got a small volume, you're really asking for trouble....We're moving to the view, we're saying that if you haven't got enough cases to meet industry norms of what's a critical minimum volume, then we're not going to pay you. We want you to get out of this business. Now that's stepping on toes, it's not very popular."

Insurers recognized that volume-outcome purchasing may be perceived as a threat to the professional autonomy of individual clinicians. One insurer noted that some medical colleges or professional associations appeared to be reluctant to specify recommended minimum volumes, while another suggested that "the challenge is actually who will be the arbiter of just what that volume will be". Another insurer suggested that volume-outcome purchasing might lead to a 'bidding war' between private hospitals to poach high profile clinicians and establish centers of excellence, as follows:

"So what you've got is the hospital operators working very much with the doctors, trying to keep them happy. So all of a sudden, gee, I'd actually like to have lobster on Friday in future. You laugh, but it actually does happen. Some doctors insist on what they're going to have provided to them for lunch while they're on their days there. And if they don't get it, no qualms about packing up and going to the next hospital. They'll take their patients with them."

Another more practical barrier to implementing volume-outcome purchasing is that data on the volume of procedures done at individual private hospitals is never publicly released by government, as this information is considered commercially sensitive. Insurers can only access such data if private hospitals voluntarily agree to provide it during contract negotiations. However, insurers noted that measurement of volume was complicated by several factors including the fact that specialists may operate at several hospitals and that some of the literature also stressed the importance of teams, not just individual clinicians, in achieving better outcomes. The impact of volume-outcome purchasing on centralizing services, with reduced geographic access in more rural locations, was also mentioned as a concern.

Finally, the potentially negative impacts on both reducing consumer choice and increasing member confusion were highlighted by this insurer.

"For instance, we've talked about what would it look like if we recommended a particular facility, you know, for maternity and another for orthopaedics. Very hard to explain to members. We think we could do that, but members still really see us and it's our responsibility is to see that we are credible as a health fund. And they essentially see us as a payer of claims, and interfering with their choice? There are ways you can structure your products and things differently, but again the product becomes much more difficult to communicate. And one of the great criticisms of the industry, and it is, is that it's complex. It is difficult to explain to members their products and the relationships with providers anyway."

#### Pay for performance

There was growing interest in the use of pay for performance models. One insurer was about to commence contract negotiations using a pay for performance model that linked payment levels to the achievement of quality standards. Several other insurers expressed interest in trialling pay for performance models in the near future. A perceived benefit was the potential of pay for performance models to promote the early adoption of practice changes such as sentinel events reporting or computerised order-entry systems for pharmaceuticals.

"Some of the things we might consider is, whether using it a bit like the Leapfrog Group, whether you can use a pay for performance approach in a sense to encourage early adoption of standards....We may look at some rewards upfront, and perhaps scaling the rewards, as it is with Leapfrog, so it's a loading of say 4% in year 1 and 2% in year 2, and sorry, it's going to become standard practice by year 3. So if you get your act together earlier, you're actually going to benefit from that."

While insurers were generally positive about the value of using financial incentives "to pursue good practice", they acknowledged that, as with volume-outcome purchasing, there was likely to be professional resistance. Moreover, it was suggested that this might result in regulatory intervention, with the government setting boundaries on what was considered to be acceptable purchasing behaviour by health insurers.

## Discussion

### Progress towards adoption of DRGs in the private sector

Even prior to the 1995 legislation enabling insurers to contract with private hospitals, there had been considerable effort invested in investigating the potential adoption of DRGs. Further to the establishment of its Casemix Development Program, the Commonwealth Government commissioned a comprehensive 1990 report that identified options for introducing DRGs in both public and private hospitals [13]. However, in 1991 the Australian Private Hospitals Association urged in relation to introducing DRGs, that the private sector should "slow down the indecent haste towards radical change to allow us to get it right the first time and avoid chaos" [14]. In 1994 private insurers, private hospitals and Commonwealth Government officials jointly developed the so-called 'Gold Book', essentially an agreed implementation guide for how casemix using AN-DRGs should be introduced into the private sector [15].

Using the Gold Book blueprint, BUPA Australia (or National Mutual Health Insurance as it was at that time) was the first private health insurer to implement an episodic case payment approach using AN-DRGs in Victoria in 1997 and South Australia in 1998. Interestingly, these two states were also the first states to adopt DRGs in the public sector. This may have facilitated uptake of case payments in private hospitals in these states, due to the familiarity of clinicians working in both public and private hospitals. Speaking at the eleventh annual casemix conference in 1999, a BUPA Australia representative indicated that the changeover process from a per diem to a DRG case payment had only been achieved by virtue of BUPA Australia mandating DRG payments in its selective tendering process for private hospital services [15]. Similarly, by 2000 MBF was able to publicly report that its competitive tendering process in Queensland had resulted in a major expansion in its use of DRGs for Queensland private hospitals [16].

This study has found that in 2004 these two companies are still the only insurers to use DRGs as the basis of purchasing most of their acute hospital services. Hence the question arises as to why the other insurers have not yet implemented DRGs, given both the 10–15 year history of developmental work and the apparent success of two leading insurers in paving the way in the private sector. The answer seems to be complex and multi-factorial, with the reasons for slow progress varying between insurers.

On the surface, the non-DRG insurers most commonly attributed the problem to concerns about the quality of private hospital cost data, claiming that the industry was historically based and that neither insurers nor private hospitals had access to adequate costing data. However, given that MBF and BUPA Australia were apparently able to overcome these problems between four to seven years ago, it is not obvious that this argument provides sufficient explanatory power. In fact, the leadership by two companies in implementing DRGs should have resulted in substantial improvement in private hospital costing data, noting that BUPA Australia and MBF have significant market shares between them in Victoria, South Australia, Queensland and New South Wales [17]. The advocacy by some private hospitals for greater uniformity in uptake of DRGs suggests that these private hospitals have confidence in their costing systems and their ability to manage in a DRG funding environment. For some of the non-DRG insurers, there seemed to be a 'chicken and egg' problem with their lack of confidence in private hospital cost data related to their lack of internal experience in working with and analysing DRG data. In contrast, BUPA Australia [15,18,19] and MBF [16,20-22] have regularly documented at annual casemix conferences their significant investment in casemix development.

In 1998 the Australian Health Insurance Association, argued in relation to private sector uptake of DRGs, that there was a "culture of inertia" with both health system providers and funders being "relatively conservative by nature" [23]. This explanation continued to ring true in 2004 for many (but not all) of the non-DRG insurers based on interviews for this study. The culture of these insurers appeared to be inherently conservative and risk averse, as reflected in their general approach to purchasing. For example, several insurers spoke of the challenges in getting support from their Boards for major changes to their purchasing frameworks. Some of these non-DRG insurers also seemed to be less confident of their ability to use their market power in driving payment model reforms, often referring to the countervailing power of both private hospitals and medical professionals.

In relation to market power, it is of interest to note that both BUPA Australia and MBF introduced DRGs in the context of competitive tendering processes. Selective contracting represents a major shift in the power dynamics between insurers and private hospitals, unleashing the power of insurers to be purchasers rather than 'passive bill-payers'. One insurer was clearly uncomfortable with this use of market power, commenting that the BUPA Australia and MBF tenders had generated "a lot of angst and a lot of dislike". However, another insurer welcomed their role in pioneering change across the industry, noting that BUPA Australia was well-regarded for its commercial adeptness and MBF was viewed as a strong leader in purchasing for quality.

### Role of current payment models in 'buying best value health care'

Based on the continuum of payment systems featured in Figure 1, the assumption behind this study is that greater use of DRGs would enable health insurers to be more effective in 'buying best value health care'. From a technical efficiency perspective, the advantages of DRG case payments over charging fees for individual items or per diem rates are well accepted. For example, the OECD in its most recent review of high-performing health systems has argued that prospective, case-related payment systems offer significant benefits in inducing hospitals to seek productivity improvements [24].

However it is evident from this study that the payment models used by most Australian private health insurers represent relatively unsophisticated approaches to purchasing. In addition to most insurers not using DRG case payments, innovation in developing new purchasing models beyond casemix was largely concentrated in one major insurer, BUPA Australia. The culture of conservatism seemed to limit the willingness of some insurers to engage in learning through implementation of payment model reforms. In 1997 a BUPA Australia representative argued in relation to the development of new funding models for rehabilitation services that:

*"The expectation is that we will learn as we proceed. We are unlikely to learn as much by standing still." *[18]

In 2004 this spirit of learning, including learning through making mistakes, did not appear to be a strongly defining characteristic of many private health insurers.

Another interpretation of the findings of this study is that health insurers view their purchasing role as having to balance competing objectives, only one of which is about improvements in technical efficiency. Insured consumers may consider that 'best value' incorporates access to a wide range of hospital services at the time of their choosing, assurances about the quality and safety of health services, and no intervention by their insurer to limit health service choices made by the patient or their doctor. As reported earlier in this paper, health insurers frequently referred to the challenges of maintaining the value proposition of health insurance. More specifically, the use of consumer co-payments, selective contracting of private hospitals and volume-outcome purchasing were viewed by some insurers as potentially reducing the value of private health insurance.

### Relationship between regulation and purchasing

This study highlighted some of the frustrations experienced by insurers in moving to a more proactive purchasing role, encapsulated in the complaint about "legislation that actually prevents you from doing a good job of purchasing". Examples of regulation viewed as problematic by insurers included the requirement that insurers pay second-tier default benefits to non-contracted hospitals, cumbersome approval processes for purchasing outreach services and the impact of reinsurance in inhibiting substitution between hospital and community-based services.

However, it is not uncommon for industry generally to use regulation as a convenient scapegoat to justify public concerns about its performance. If regulation were really a significant barrier to improved purchasing behavior by private health insurers, it would be expected that it would impact equally on all health insurance companies. Instead, Australian health insurers exposed to an identical regulatory environment have developed very different approaches to purchasing health services for their members. The innovative purchasing models used by BUPA were developed despite the negative impacts through reinsurance arrangements for BUPA of some of these models.

While it is clearly desirable for regulation to promote effectiveness and efficiency in purchasing so that insured people get 'the best care for the best price', the regulatory environment is not the only factor influencing purchasing behavior. In particular, regulation cannot trump institutional history and culture, nor can it create savvy, entrepreneurial insurers that are skilful in parlaying relationships with health service providers into best value health care for their members.

The relationship between regulation and effective purchasing is complex. Most commentators in the health arena recognize the need for a significant role for government in regulating health markets due to well accepted market failures such as information asymmetry, externalities and the potential for supplier induced demand [25]. It has previously been noted that the *National Health Act 1953 *lacks clear and coherent policy objectives that explain the purpose of government regulation of this industry. The theoretical rationale for government intervention can include promoting consumer access to affordable and attractive products, improving accountability to consumers, encouraging competition and efficiency in the private health sector, and promoting the financial sustainability of the sector [26]. Insurers noted the tension in balancing some of these objectives, including the trade-off between broad choice of providers and services, low co-payments and affordable health insurance premiums.

While the first priority must be greater clarity about the policy objectives behind private health insurance regulation, the regulatory framework for purchasing could also be improved. The approval process for outreach services is an example of a detailed command-and-control approach to regulation that would no longer be regarded as best practice [27]. A preferred approach to private health insurance regulation generally would be greater adoption of performance-based regulation. This would clearly specify the desired outcomes but allow private health insurers the opportunity to be flexible and innovative in how they achieve these outcomes [28].

## Conclusion

Since the passage of the 1995 legislation allowing health insurers to take a more pro-active role in purchasing, health insurers have sought to balance the competing demands of private hospitals, medical professionals, consumers and government. While health insurers see the relationship with private hospitals as symbiotic, some have concerns about the potential for unnecessary medical servicing and the lack of transparency on private hospital costs. There are also mixed views about the merits of pursuing a more aggressive policy on selective contracting of private hospitals, notwithstanding that competitive tendering has allowed two major insurers to implement more sophisticated payment models. Progress towards greater adoption of case payments using DRGs has been much slower than anticipated. Industry conservatism and resistance to change, coupled with inadequate investment in hospital cost analysis, may have contributed to this situation. However, the foreshadowed move by two other major insurers to base their purchasing strategies on DRGs, together with the growing demand from private hospitals for uniformity in payment models, may finally swing the pendulum towards more effective purchasing of best value care.

## Competing interests

The author(s) declare that they have no competing interests.

## Supplementary Material

Additional File 1 Table 1, which shows 4 different payment models (e.g. Interim care, EMU) in use by one insurer. Click here for file
