# Supplementary material for: Buying best value health care: Evolution of purchasing among Australian private health insurers
Source: Aust New Zealand Health Policy. 2005 Mar 31;2:6. doi: 10.1186/1743-8462-2-6 (PMC1079790; doi:10.1186/1743-8462-2-6)
Supplement: Additional File 1 — Table 1, which shows 4 different payment models (e.g. Interim care, EMU) in use by one insurer. [file 1743-8462-2-6-S1.doc]

**Table 1: Innovative payment models developed by one health insurer**

| **Payment model** | **Features of the model** | **Advantages and outcomes of the model** |
| --- | --- | --- |
| Interim care program | Insurer pays for patient to be accommodated in a transitional setting between hospital and home through a contract with a third party (non-hospital) agency; Insurer encourages hospitals to use these services through contract requirements on use of accredited discharge services | - Independent agency assesses readiness of patients to go home including need for low-level support services and arranges transfer of patients - Frees capacity in hospitals for patients who need more acute care - Has created a stronger focus on non-medical needs and skills required by patients (particularly older patients) so they can safely return home |
| Episodic Management Unit (EMU) | Hospital receives a single payment explicitly including necessary rehabilitation for defined services (such as heart surgery or hip replacements); similar to an "expanded DRG" | - Reduces the risk of patients falling between service gaps as hospital takes responsibility for care plan across total episode - Has promoted expansion of community-based rehabilitation programs - Has reduced dependence on inpatient rehabilitation and previous practice of many patients being routinely transferred to a rehabilitation hospital |
| Members Extended Care Arrangement (MECA) | Hospital receives a single payment that includes responsibility for any re-admissions or transfers to another hospital within 14 days of discharge | - Reduces risk of premature discharge by hospitals as they become responsible for subsequent readmission costs - Has created stronger focus on care plans and discharge planning services |
| Capitation for psychiatric services | Insurer pays a provider a monthly payment based on the number of insured members to provide all necessary psychiatric service | - Has resulted in a major expansion in outpatient programs - Has resulted in greater focus on specifying desired outcome and quality parameters |
